# Targeting the UPR with Small Molecules: Emerging Strategies for Immune Regulation

**DOI:** 10.3390/molecules31030559

**Published:** 2026-02-05

**Authors:** Junyi Duan, Daoyuan Huang, Yick W. Fong

**Affiliations:** 1 Brigham Regenerative Medicine Center, Brigham and Women’s Hospital, Boston, MA 02215, USA; 2 Division of Cardiovascular Medicine, Heart and Vascular Institute, Department of Medicine, Harvard Medical School, Boston, MA 02215, USA; 3 Harvard Stem Cell Institute, Cambridge, MA 02138, USA; 4 Department of Pathology, Beth Israel Deaconess Medical Center, Harvard Medical School, Boston, MA 02215, USA

**Keywords:** ER, UPR, small molecules, immunity, SG, LCD

## Abstract

The unfolded protein response (UPR) is a highly conserved adaptive mechanism that restores endoplasmic reticulum (ER) homeostasis under stress. Beyond its canonical roles in proteostasis, the UPR has emerged as a central regulator of immune responses across diverse contexts, including infection, inflammation, cancer, and autoimmunity. IRE1α, PERK, and ATF6 are three principal UPR sensors that coordinate complex signaling networks to regulate antigen presentation, cytokine production, and immune cell differentiation. This review highlights the molecular mechanisms by which small molecules target the UPR to modulate immune responses. In addition, we highlight stress granules (SGs) and the prevalence of protein–protein interactions mediated by intrinsically low-complexity domains (LCDs) in the UPR as potential new avenues for immune modulation. Finally, we discuss future directions for leveraging UPR modulation in immunotherapy, infectious disease, and chronic inflammatory disorders.

## 1. The UPR and Its ER Stress Sensors

**Figure 1:**
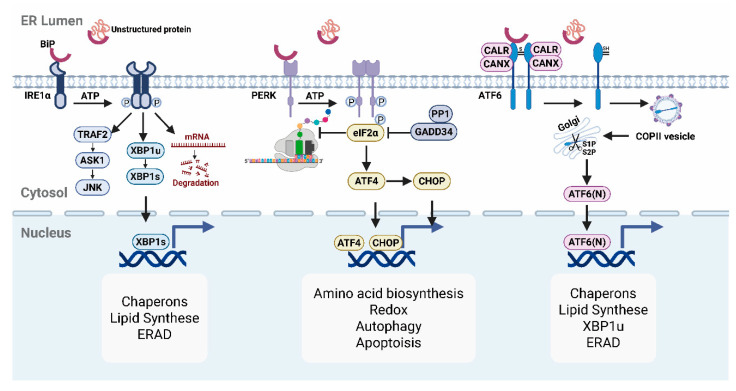
Overview of the three canonical UPR branches. Upon accumulation of unfolded proteins in the ER lumen, the chaperone BiP dissociates from the ER stress sensors IRE1α, PERK, and ATF6, triggering their activation. IRE1α oligomerizes and autophosphorylates, initiating unconventional *XBP1* mRNA splicing to generate XBP1s. XBP1s in turn upregulate chaperones, lipid synthesis, and ERAD genes. Under prolonged stress, IRE1α also mediates RIDD-dependent mRNA decay and TRAF2–JNK signaling, linking ER stress to apoptosis. PERK phosphorylates eIF2α, attenuating global translation while selectively enhancing ATF4 translation to regulate amino acid metabolism, redox balance, and autophagy. Persistent CHOP activation shifts the response toward apoptosis. In response to ER stress, ATF6 translocates to the Golgi, where it undergoes S1P- and S2P-mediated proteolysis to release the active ATF6(N) fragment. This fragment then enters the nucleus to induce the expression of chaperone and ERAD genes.

Environmental insults, pathological and genetic factors, or cellular senescence can elevate protein-folding stress in the ER. To restore proteostasis in cells, the ER initiates the UPR [1]. In mammals, the UPR operates through three ER-bound transmembrane sensors, IRE1α, PERK, and ATF6, which activate sensor-specific signaling that allows the ER to adjust its functional capacity under stress (Figure 1).

### 1.1. IRE1α Pathway: The Most Conserved UPR Branch

Among the three canonical branches of the UPR, IRE1α pathway is the most evolutionarily conserved, spanning from yeast to human [2]. IRE1α is a transmembrane sensor protein characterized by a luminal stress-sensing domain and a cytosolic kinase/endoribonuclease (RNase) domain. Under homeostatic conditions, IRE1α remains in an inactive monomeric state through its association with the chaperone BiP [3]. Upon accumulation of unfolded proteins in the ER lumen, BiP dissociates from IRE1α, which allows IRE1α to undergo oligomerization and trans-autophosphorylation, triggering its downstream signaling functions.

Activated IRE1α executes its functions through three distinct mechanisms. First, it catalyzes the non-canonical splicing of *XBP1* mRNA, removing a 26-nucleotide intron to generate the active transcription factor XBP1s [4]. XBP1s upregulate genes involved in ER quality control, lipid biosynthesis, and glucose metabolism [5]. Second, under prolonged or excessive ER stress, through a process known as regulated IRE1α-dependent decay (RIDD), IRE1α utilizes the same RNase activity required for *XBP1* mRNA splicing to degrade specific subsets of mRNAs and microRNAs. This helps reduce the protein-folding load within the ER [6]. Third, when ER stress cannot be resolved, IRE1α recruits TRAF2 to activate the JNK (c-Jun N-terminal kinase) pathway, thereby promoting pro-apoptotic signaling and cell death [7].

### 1.2. PERK Pathway: Translation Attenuation Under Stress

Structurally similar to IRE1α, PERK harbors a luminal stress-sensing domain and a cytosolic serine/threonine kinase domain. Upon accumulation of misfolded proteins, BiP dissociates from PERK, triggering its dimerization and autophosphorylation [3]. The primary role of PERK is to modulate global translation. PERK-mediated phosphorylation of eIF2α at Ser51 inhibits global mRNA translation initiation by preventing eIF2B-mediated GTP exchange, which is essential for cap-dependent translation. This reduces the influx of newly synthesized proteins into the ER. However, translation of transcripts containing upstream open reading frames (uORFs) is selectively enhanced, including ATF4, which regulates genes involved in antioxidant, amino acid biosynthesis, and mitochondrial function [8]. One of its key downstream effectors, CHOP, initially contributes to adaptive responses by inducing GADD34, which promotes eIF2α dephosphorylation and translation recovery [9]. However, under conditions of chronic or irreversible ER stress, sustained CHOP expression promotes apoptosis by inducing pro-apoptotic genes such as Bim and DR5, disrupting mitochondrial function, and increasing reactive oxygen species (ROS) production [10].

It is worth noting that PERK is one of several eIF2α kinases. Stress sensors such as PKR, GCN2, and HRI can also phosphorylate eIF2a in response to distinct cellular stresses and contribute to the integrated stress response (ISR) [11], highlighting the context-specific roles of PERK in ER homeostasis.

### 1.3. ATF6 Pathway: A Transcriptional Regulator of ER Proteostasis

Distinct from IRE1α and PERK (type I), the ATF6 is a type II ER transmembrane transcription factor precursor with a cytosolic bZIP domain. Under normal conditions, ATF6 forms disulfide-linked oligomers in the ER and associates with BiP. BiP masks ATF6’s Golgi localization signals (GLSs), thereby preventing its premature translocation to the Golgi, a necessary step for ATF6 activation [3]. Upon ER stress, unfolded proteins sequester BiP away from ATF6. Through unmasking its GLSs and PDI-mediated disulfide reduction, monomeric ATF6 is trafficked to the Golgi [12]. At Golgi, ATF6 is sequentially cleaved by S1P and S2P proteases, releasing the transcriptionally active, cytosolic ATF6(N) fragment. ATF6(N) then translocases into the nucleus and activates its target genes [13]. Compared to IRE1α and PERK, ATF6 appears to play a more prominent role in enhancing protein folding and clearance capacity of the ER by upregulating chaperones (e.g., BiP, GRP94) and ER-associated degradation (ERAD) component (e.g., *EDEM1*) genes [14,15]. Unlike IRE1α and PERK branches, ATF6 is less involved in apoptotic or translational control, but favors adaptive remodeling of the ER.

### 1.4. ER Stress Recovery Pathways

Cells rely on a multifaceted protein quality control system to restore ER homeostasis under stress. Protein chaperones, such as BiP, GRP94, and GRP170, promote proper protein folding by recognizing and stabilizing unfolded or misfolded polypeptides [16]. In parallel, lectin chaperones calnexin and calreticulin assist glycoprotein folding through iterative cycles involving glucose trimming and re-glycosylation [17]. Folding catalysts, including protein disulfide isomerases (PDIs) and peptidyl-prolyl isomerases (PPIs), further ensure correct protein folding through regulated disulfide bond formation and proline isomerization [18,19]. When these fail-safe mechanisms fail, misfolded proteins are targeted for degradation via ERAD pathway involving the ubiquitin–proteasome system [20]. For aggregation-prone or ERAD-resistant proteins, ER-phagy, a selective form of autophagy [21], mediates their removal through lysosomal degradation.

## 2. The Role of the UPR in Immune Homeostasis and Regulation

Although UPR is traditionally viewed as an adaptive response to ER stress, increasing evidence indicates that UPR signaling also operates under homeostatic and pathological conditions. In this section, we summarize the roles of the UPR in regulating immune cell function and identity, and how dysregulation of the UPR contributes to pathological immune states.

### 2.1. Basal Activity of the UPR in Immune Cells

ER stress and UPR are not activated only under pathological conditions in the immune system. Rather, UPR signaling exhibits constitutive activity in specific immune cell types. Using IRE1α activity reporter systems (e.g., ERAI), persistent activation of the IRE1α–XBP1 signaling axis has been observed in splenic conventional dendritic cells (cDCs), plasmacytoid dendritic cells (pDCs), macrophages, and natural killer (NK) cells under steady-state conditions [22]. This basal activity is closely associated with the increased demands of these cells for protein synthesis, antigen processing, and cytokine secretion. In contrast, naive T cells, B cells, monocytes, and neutrophils exhibit relatively low basal UPR activity [23,24], highlighting cell-type-specific differences in UPR activities. Moreover, the UPR is dynamically regulated throughout immune cell development. For instance, in CD8^+^ T cell maturation, IRE1α is selectively activated during the transition from the CD4^−^CD8^−^ stage to the CD4^+^CD8^+^ stage to support T cell receptor (TCR) synthesis [25]. In mature CD8^+^ T cells, high IRE1α activity is maintained during immune responses to facilitate lipid metabolism, protein glycosylation, and the expression of effector molecules such as KLRG1, granzyme B, and perforin [26,27].

These context- and lineage-specific UPR activation profiles not only reflect the functional heterogeneity of immune cells but also provide a foundation for selective targeting of UPR pathways for immune modulation, minimizing the adverse effects of systemic inhibition.

### 2.2. UPR-Mediated Regulation of Immune Function

**Figure 2:**
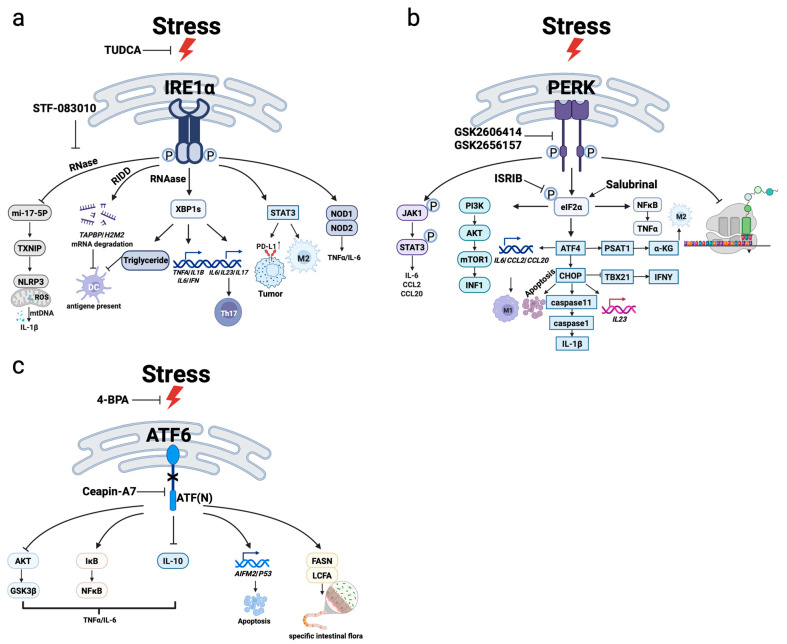
UPR signaling orchestrates immune regulation under ER stress. (**a**) Upon activation, IRE1α promotes XBP1s-dependent transcription of cytokines and enhances Th17 differentiation, antigen presentation, and plasma cell maturation. Concurrently, RIDD activity degrades select mRNAs that impair antigen cross-presentation in dendritic cells, while TRAF2/ASK1–JNK signaling and TXNIP–NLRP3 inflammasome activation link ER stress to proinflammatory responses. (**b**) PERK activation induces ATF4/CHOP-driven transcription of *IL-6*, *CCL2*, and *CCL20*, promoting M1 polarization and neuroinflammation. PERK also interfaces with JAK1–STAT3, NF-κB, and mTOR pathways, modulating cytokine release and T cell fates through selective translational and apoptosis regulation. (**c**) Activated ATF6(N) augments NF-κB-mediated IL-6/TNFα expression while repressing AKT–GSK3β signaling and IL-10 production. ATF6 enhances FASN-mediated LCFA synthesis, shaping intestinal microbiota and inflammation.

Three UPR canonical signaling branches—IRE1α, PERK, and ATF6—are intricately involved in immune signaling beyond protein folding control, by contributing to cytokine regulation, antigen presentation, and broader immune cell function (Figure 2).

#### 2.2.1. IRE1α–XBP1 Pathway: A Central Integrator of Immune Regulation

As an innate immune response, IRE1α–XBP1 signaling modulates inflammasome activation, Toll-like receptor (TLR) signaling, and proinflammatory cytokine production. Mechanistically, IRE1α promotes mitochondrial ROS generation and miR-17-5p degradation, thereby upregulating TXNIP to activate the NLRP3 inflammasome and enhance IL-1β secretion [28,29]. In addition, TLR4 stimulation by LPS triggers ROS and TRAF6-mediated activation of IRE1α, which in turn promotes XBP1s-driven transcription of *TNF-α*, *IL-1β*, and *IL-6* [30].

More importantly, the IRE1α–XBP1 axis plays essential roles in adaptive immunity. In T cells, hypoxic and low-glucose conditions activate the IRE1α–XBP1 signaling axis, which drives the expression of Th17-associated effector cytokines (IL-6, IL-1β, and TGF-β). This activation promotes Th17 cell differentiation and reinforces their effector functions [31]. In B cells, mTORC1 signaling induces the upregulation of XBP1s, a master regulator of plasma cell differentiation and antibody secretion efficiency by maintaining the expanded ER network and facilitating proper immunoglobulin folding [32,33].

Additionally, IRE1α regulates antigen presentation through RIDD. In DCs, IRE1α remains constitutively active even in the absence of overt ER stress. Under XBP1 deficiency, compensatory activation of RIDD by IRE1α leads to the degradation of mRNAs encoding MHC class I peptide-loading complex components, such as *Tapbp* and *H2-M2* [24], thereby impairing cross-presentation capacity.

Within the tumor microenvironment (TME), aberrant accumulation of lipid peroxides triggers IRE1α–XBP1 activation, which further disrupts lipid metabolic homeostasis [34]. The excessive lipid deposition hampers effective antigen presentation, weakens CD8^+^ T cell activation, and ultimately contributes to immune evasion.

Together, the IRE1α–XBP1 pathway not only functions as a stress response mechanism but also as a key coordinator of immune sensing, inflammation, and adaptive immune programming.

#### 2.2.2. PERK–eIF2α-ATF4–CHOP Pathway: A Stress Switch to Immune Activation and Fate Decisions

The PERK pathway is rapidly activated in response to acute stress. In the experimental autoimmune encephalomyelitis (EAE) mouse model, PERK has been shown to interact with JAK1, leading to JAK1 phosphorylation and subsequent activation of STAT3 signaling, thereby promoting IL-6 secretion and neuroinflammatory demyelination [35]. Similarly, activation of ATF4 enhances transcription of proinflammatory mediators such as *IL-6*, *CCL2*, and *CCL20* in glial cells, driving their transition toward an M1-like proinflammatory phenotype and contributing to chronic neuroinflammation [36,37]. The downstream effector of ATF4, CHOP, can upregulate IL-23 and IL-1β secretion, thereby facilitating neutrophil recruitment during pathogen invasion [38,39]. Notably, PERK does not always mediate inflammation through the canonical ATF4/CHOP axis. For example, STING activation induces a conformational change that allows for direct interaction with PERK, promoting its autophosphorylation and subsequent activation of eIF2α. This establishes a proinflammatory translational program that enhances the ribosomal association of TNF-α- and NF-κB-related mRNAs, without significant upregulation of ATF4 or CHOP [40].

PERK signaling also regulates the T cell immune response cycle. The immune cell response cycle includes activation, proliferation, and resolution phases [41]. During the contraction phase of immune responses, CHOP-mediated stress drives apoptosis of activated T cells to prevent excessive inflammation. Conversely, moderate PERK activity supports memory T cell formation and survival [42]. Notably, PERK-induced translational reprogramming may generate noncanonical or “cryptic” antigens through initiation at alternative codons (e.g., CUG) [43], increasing antigen diversity—a concept relevant for vaccine design and immune escape mechanisms.

In summary, the PERK pathway acts as a dynamic “stress switch” in immune cells, and the degree and timing of its activation dictate the immune response outcome.

#### 2.2.3. ATF6 Pathway: A Potential Modulator of Immune Coordination

Compared to IRE1α and PERK, the ATF6 branch is less well-studied in immunology. ATF6α is more responsive to ER stress than its isoform ATF6β and may function to fine-tune immune responses during early stress phases. In bone marrow-derived macrophages, ATF6 activation enhances NF-κB signaling and inhibits the AKT/GSK3β axis, thereby amplifying TLR4-driven inflammation by increasing IL-6 and TNF-α expression and reducing IL-10 induction [44]. ATF6 also plays a pivotal role in regulating the gut microbiota. In colorectal cancer (CRC), ATF6 activation markedly upregulates fatty acid synthase (*FASN*), leading to elevated levels of long-chain fatty acids (LCFAs) in both colonic epithelial cells and the intestinal lumen. This alteration in the intestinal metabolic milieu selectively enriches tumor-associated sulfate-reducing bacteria (SRB), thereby reshaping the gut microbial ecosystem in favor of tumor progression [45].

In pancreatitis models, premature trypsinogen activation induces ATF6, which directly binds to the *P53* and *AIFM2* promoters to upregulate IL-6, IL-1β, and TNF-α, resulting in pancreatic injury [46]. In addition to regulating classical targets such as *EDEM1* and *PDIA6*, ATF6 has also been shown to enhance *XBP*1 mRNA splicing [47], suggesting functional synergy with the IRE1α pathway. Though the immunological roles of ATF6 are still being defined, ATF6 represents a potentially expandable target in future therapeutic strategies.

### 2.3. UPR Dysregulation and Immune Imbalance in Pathological States

Under pathological stimuli, persistent or aberrant UPR activation often disrupts this equilibrium, leading to immune dysfunction. Such dysregulation is prominently involved in tumor-associated immunosuppression, autoimmune diseases, and viral infections.

#### 2.3.1. UPR-Mediated Immunosuppression and Tumor Immune Evasion

In the TME, metabolic stressors such as hypoxia, ROS, and lactate accumulation increase ER burden in immune cells, triggering sustained UPR activation. In this context, the UPR contributes to the establishment of an immunosuppressive state, particularly within tumor-associated macrophages (TAMs), tumor-infiltrating lymphocytes (TILs), and myeloid-derived suppressor cells (MDSCs) [48].

The IRE1α–XBP1 signaling axis is highly active in TAMs, where it reprograms lipid metabolism and upregulates immunosuppressive gene expression. This drives polarization toward an M2-like phenotype, facilitating immune evasion and tumor progression [49]. In addition, viruses such as Kaposi’s sarcoma-associated herpesvirus (KSHV) can activate IRE1α in TAMs. This activation enhances the release of tumor-promoting cytokines and upregulates PD-L1 expression, thereby enabling tumors to evade immune surveillance [50].

In TILs, intracellular cholesterol accumulation has also been shown to activate XBP1s, which induces the expression of inhibitory receptors such as PD-1 and TIM-3, facilitating T cell exhaustion and impairing cytotoxic function [51].

The PERK pathway also plays a critical role in promoting immune suppression within the TME. In both TAMs and TILs, ATF4 and CHOP interfere with mitochondrial metabolism and suppress T-bet expression, thereby reducing IFN-γ production and weakening CD8^+^ T cell effector responses [52]. Moreover, PERK–ATF4 signaling upregulates *PSAT1*, an enzyme of the serine biosynthesis pathway. The resulting accumulation of α-ketoglutarate (α-KG) activates the histone demethylase JMJD3, driving M2-specific epigenetic reprogramming and immunosuppressive phenotype [53,54].

ATF6 has also been implicated in the specification of immunosuppressive lineages. In polymorphonuclear MDSCs (PMN-MDSCs), ATF6 activation is essential for the acquisition of suppressive function [55]. Loss of ATF6 in these cells enhances antigen-specific T cell responses and delays tumor progression.

#### 2.3.2. Aberrant UPR Activation in Autoimmune Diseases

In contrast to its immunosuppressive role in tumors, UPR activation in autoimmune diseases is often proinflammatory and contributes to disease pathogenesis.

In rheumatoid arthritis (RA), serum levels of UPR-associated chaperones such as GRP78 and GRP94 are elevated. These proteins can induce autoantibody production and enhance inflammatory cytokine release [56]. Additionally, metabolic perturbations, such as aspartate depletion, can disrupt the mitochondrial NAD^+^/NADH ratio, leading to IRE1α activation and amplification of inflammatory responses [57].

In type 1 diabetes (T1D), IL-1β and TNF-α released by infiltrating CD8^+^ T cells trigger severe ER stress in pancreatic β cells [58]. IRE1α promotes β cell apoptosis through both TRAF2–JNK–CHOP pathway and NF-κB-mediated cytokine release [59]. PERK can also promote β cell death through converging on CHOP [60], while hyperactivation of ATF6 suppresses insulin gene transcription, further contributing to β cell dysfunction [61].

In inflammatory bowel disease (IBD), NOD2 sensing of bacterial components couples innate immune signaling to the ER via LACC1. This interaction promotes the activation of all three ER stress branches—PERK, IRE1α, and ATF6—triggering MAPK and NF-κB signaling in macrophages and driving proinflammatory cytokine production and bacterial clearance [62]. Disease-associated mutations in LACC1 disrupt this ER stress-dependent signaling network, leading to impaired barrier integrity.

Metabolic stressors, such as saturated fatty acid accumulation, can activate mitochondrial ROS production and XBP1–TLR pathways in dendritic cells, driving IL-23 secretion and exacerbating psoriasis-like skin inflammation [63]. Furthermore, elevated XBP1s expression has been observed in peripheral blood mononuclear cells of patients with vitiligo, suggesting a potential role of IRE1α in immune tolerance.

#### 2.3.3. UPR Reprogramming and Immune Interference During Viral Infections

During viral infection, the UPR serves as a host defense mechanism. However, viruses have developed elaborate mechanisms to manipulate the UPR to facilitate viral replication and immune evasion.

Many RNA viruses, including members of the Flaviviridae family (e.g., HCV, DENV, WNV) and coronaviruses (e.g., SARS-CoV), rely on the host ER as a replication platform. These viruses induce the UPR to remodel ER membranes and enhance viral protein folding. For example, HCV and DENV activate the IRE1α–XBP1axis to upregulate ER chaperones such as BiP to enhance viral protein production [64]. The SARS-CoV accessory protein 8ab directly interacts with ATF6, thereby inducing its activation, promoting ER membrane biogenesis to facilitate viral particle assembly.

Conversely, some viruses suppress the PERK pathway to block premature apoptosis (e.g., WNV), or modulate eIF2α phosphorylation levels (e.g., HBV) to reduce viral protein synthesis to promote latency and immune evasion [65].

The RIDD activity of IRE1α can generate immunostimulatory RNA fragments that act as danger signals to initiate antiviral responses [66]. Viruses such as CMV suppress RIDD-mediated antiviral signaling by expressing proteins, such as M50, that target IRE1α for ERAD-mediated degradation [67]. Interestingly, viruses that replicate in the ER (e.g., HCV, JEV, CMV) may activate RIDD at a moderate level to fine-tune the translational landscape, promoting chronic infection and immune evasion without triggering host cell death [68].

## 3. Small-Molecule Modulators Targeting UPR Proteins

IRE1α, PERK, and ATF6 possess distinct structural and functional domains, including RNase domains, oligomerization regions, ATP-binding sites, and interaction interfaces for chaperones and downstream effectors. Small molecules targeting these domains have been developed to modulate UPR signaling pathways and have demonstrated therapeutic potential in models of tumor immunity and infectious and autoimmune diseases (Table 1 and Table 2).

### 3.1. Modulators Targeting the RNase Domain of IRE1α

The RNase domain of IRE1α plays a key role in regulating the physiological outcome of the UPR. In various inflammatory diseases, IRE1α’s RNase activity is often pathologically activated, driving the expression of pro-inflammatory cytokines and the hyperactivation of effector T cells. This domain mediates the unconventional splicing of *XBP1* mRNA into its active form, XBP1s, a transcription factor that regulates numerous immune-related genes.

Salicylaldehyde derivatives such as 3-ethoxy-5,6-dibromosalicylaldehyde and 3-methoxy-6-bromosalicylaldehyde were among the first small-molecule inhibitors identified to selectively block IRE1α RNase activity [69]. These compounds bind noncompetitively to IRE1α and potently inhibit *XBP1* mRNA splicing, thereby attenuating downstream XBP1s-directed transcriptional programs that promote influenza A virus (IAV) protein synthesis and viral replication [88].

IRE1α is activated in lipid-laden macrophages (foam cells) in atherosclerotic lesion sites. Ozlem et al. found that STF-083010, a direct inhibitor of the RNase domain of IRE1α, significantly reduces lipid-induced activation of the NLRP3 inflammasome and secretion of pro-inflammatory cytokines IL-1β and IL-18 as well as the chemokine CCL2, and mitigates inflammation and atherosclerotic plaque size in hyperlipidemic mouse models [77]. Similarly, in hypoxia–ischemia (HI) injury models, STF-083010 not only suppresses the inflammatory response but also significantly reduces infarct size and improves neurological outcomes [29]. Within the melanoma TME, elevated cholesterol levels induce ER stress in CD8^+^ T cells, activating XBP1 and upregulating immune checkpoint molecules such as PD-1 and 2B4, thereby promoting T cell exhaustion and tumor immune evasion. Inhibiting *XBP1* mRNA splicing using STF-083010 or reducing intracellular cholesterol restores T cell effector function and markedly enhances anti-tumor immunity [51]. Viral single-stranded RNA can also activate IRE1α RNase activity via TLRs (TLR7/8), leading to *XBP1* mRNA splicing. MKC3946, another IRE1α RNase inhibitor, has shown efficacy in mitigating immune hyperactivation and cytokine storms in models of SARS-CoV-2-induced pneumonia [76]. Agonists of IRE1α RNase activity are also available. IXA4 induces XBP1s splicing without initiating RIDD, thus restoring mitochondrial function and metabolic capacity in pDCs [72]. In obese mouse models, IXA4 improves insulin secretion and glucose homeostasis, illustrating the tight coupling of UPR signaling and immunometabolism [89]. This class of compounds highlights the therapeutic potential at the immunometabolic junction, particularly for chronic inflammatory metabolic syndromes such as obesity, diabetes, and non-alcoholic fatty liver disease (NAFLD).

### 3.2. Modulators Targeting Kinase and ATP-Binding Domains of UPR Sensors

Autophosphorylation by the kinase domain of IRE1α is pivotal for activating its RNase activity. Small molecules that inhibit this domain can thus attenuate downstream signaling.

KIRA6 and KIRA8 are prototypical kinase-inhibiting RNase attenuators (KIRAs), which competitively block the ATP-binding site in IRE1α, thereby preventing IRE1α autophosphorylation and RNase activation. In inflammatory models, KIRA6 has been shown to significantly suppress IRE1α-dependent IL-1β production and inhibit excessive inflammatory responses triggered by Citrobacter rodentium infection [73]. Similarly, KIRA8 has demonstrated efficacy in Type 1 diabetes models by protecting β-cells from autoimmune T cell-mediated destruction [74], suggesting that targeting the kinase domain can help preserve immune tolerance in tissues.

Other ATP-competitive inhibitors, such as staurosporine and sunitinib, have also been shown to bind the ATP-binding pocket of IRE1α, thereby blocking autophosphorylation and activation of its RNase module. This suppression effectively impedes multiple myeloma progression [90]. However, these compounds function as broad-spectrum kinase inhibitors and thus lack target specificity toward IRE1α.

GSK2606414 and GSK2656157 are ATP-competitive inhibitors that target the PERK kinase domain, blocking its autophosphorylation and downstream signaling.

PERK signaling is activated in tumor-associated myeloid-derived suppressor cells (MDSCs), where it maintains NRF2-driven mitochondrial homeostasis and antioxidant capacity, thus promoting immunosuppressive function of MDSCs by preventing mtDNA leakage and pro-inflammatory by STING activation. Inhibition of PERK by GSK2606414 promotes a STING-dependent IFN-γ response, which can overcome immunosuppression [79]. GSK2656157 has been shown to attenuate macrophage immunosuppressive activity and enhance the efficacy of PD-1 immune checkpoint blockade [53]. Compared to these earlier compounds, AMG44 offers improved selectivity, pharmacokinetics, and safety. It also binds competitively to the PERK kinase domain and inhibits autophosphorylation. PERK activation promotes the expansion of inflammatory Th1 and Th17 while reducing Treg differentiation via activation of the NRF2 pathway, thereby contributing to graft-versus-host disease (GVHD) [78]. AMG44 effectively suppresses GVHD induced by T cells in humans and mice through targeted PERK inhibition

### 3.3. Modulators Targeting the Oligomerization Domains of UPR Sensors

Initiation of UPR signaling largely depends on the oligomerization of ER sensor proteins. Under ER stress, IRE1α, PERK, and ATF6 undergo conformational changes that promote oligomer formation, which is essential for activating their cytosolic effector domains. Therefore, inhibiting this oligomerization process may have additional benefits of more effectively suppressing the UPR at an early stage.

Recent studies have shown that the AKT–mTOR pathway dynamically inhibits IRE1α oligomerization and RNase activity by regulating ER–mitochondria contact sites. Inhibition of AKT (MK2206) or mTOR (rapamycin) disrupts the spatial clustering of IRE1α, paradoxically resulting in hyperactivation of RNase activity, leading to inflammatory gene expression and cell death [91].

In vitro studies further reveal contrasting effects of kinase inhibitors on IRE1α oligomerization. When incubated with IRE1α proteins, APY29 increases the oligomer-to-monomer ratio of IRE1α in a concentration-dependent manner, while Compound **3** reduces this ratio. These two kinase inhibitors exhibit opposing effects on IRE1α RNase activity, likely by influencing the enzyme’s oligomeric state [70]. Although APY29 activates the RNase function of IRE1α by inducing a conformational change, it is still classified as a kinase inhibitor of IRE1α, because it binds competitively to the ATP-binding site of the kinase domain. As a result, APY29 inhibits IRE1α phosphorylation to promote macrophage polarization from a proinflammatory M1 phenotype toward an anti-inflammatory M2 phenotype and suppresses the expression of inflammatory cytokines such as TNF-α and IL-6 [92].

### 3.4. Modulators Targeting Protein–Protein Interaction Interfaces

UPR sensor activation is also regulated by protein–protein interactions (PPIs), particularly with molecular chaperones like BiP, which maintain sensors in an inactive state during homeostasis. Small molecules that target these protein interaction surfaces allow for fine-tuning of UPR activity by modulating sensor activation. Small-molecule agonist HA15 promotes the release of BiP from IRE1α, thereby activating IRE1α signaling. HA15-induced activation of IRE1α enhances MHC-I-dependent antigen processing and presentation by dendritic cells, increases antigen-specific T cell activation, and boosts the immunogenicity of tumor cells [71].

Kaempferol, a natural flavonoid, alleviates ER stress in bronchial epithelium and reduces asthma-related mucus hypersecretion. Mechanistically, kaempferol disrupts the interaction between IRE1α and TRAF2, thereby blocking the proinflammatory effects of JNK pathway, leading to downregulation of key mucus-associated genes such as *MUC5AC* and *TGFB1* [75].

In the ATF6 pathway, Ceapin-A7 specifically inhibits ATF6 by stabilizing its complex with ER chaperones (BiP) and membrane tether proteins (CALR, CANX), preventing the trafficking of ATF6 to the Golgi apparatus for proteolytic cleavage and activation. This dampens ATF6 activation and plays a protective role in lipid metabolism-associated inflammation. Using mutant ATF6 constructs that allow for chemically induced oligomerization, studies revealed that the degree of ATF6α oligomerization positively correlates with its ER retention by Ceapins [83].

Another strategy involves serine protease inhibitors, such as AEBSF, which block the activity of S1P, a protease in the Golgi that cleaves ATF6α and ATF6β. AEBSF thereby inhibits the proteolytic activation of ATF6 and suppresses the transcriptional activation of its target genes [86]. In the context of SARS-CoV-2 infection, ATF6 is activated early during viral replication. Inhibiting ATF6 with AEBSF significantly reduces the production and release of viral particles [82].

### 3.5. Molecules Targeting UPR and Downstream Effectors

Some small-molecule UPR modulators do not directly bind canonical UPR sensors. Instead, they increase ER remodeling and protein-folding capacity, or target downstream effectors such as eIF2α phosphorylation, thereby exerting a significant influence on immune and metabolic homeostasis.

The Sigma-1 receptor (Sig-1R) is a chaperone localized at mitochondria-associated ER membranes (MAMs) and plays key roles in protein folding, calcium exchange between the ER and mitochondria, and membrane architecture stabilization. During SARS-CoV-2 infection, the viral non-structural protein Nsp6 interacts with Sig-1R to facilitate the formation of viral replication vesicles and remodeling of ER structures. Sig-1R antagonists—fluvoxamine and SA4503—can suppress CoV-induced stress and disrupt viral replication [87].

4-Phenylbutyric acid (4-PBA), a chemical chaperone, facilitates correct protein folding, thereby suppressing UPR sensor activation [93]. In allergic asthma models, 4-PBA attenuates ER stress, and exerts anti-inflammatory and immunomodulatory effects by markedly suppressing NF-κB activity, decreasing inflammatory cytokines (including IFN-γ, IL-4, IL-5, IL-13, TNF-α, IL-1β, IL-17), and reducing inflammatory cell infiltration in the lungs [84]. Persistent UPR activation is a hallmark of metabolic disorders such as obesity and type 1 and 2 diabetes. The small molecule Azoramide induces ER chaperone expression, which, in turn, improves ER protein-folding capacity and reduces ER stress, ultimately improving insulin secretion and diabetes-related phenotypes [85].

Another key molecule, TUDCA (tauroursodeoxycholic acid), alleviates ER stress by stabilizing the ER membrane structure and modulating calcium homeostasis and folding enzyme activities [94]. In tumor-infiltrating CD8^+^ T cells, ER stress-induced CHOP upregulation suppresses the transcription factor T-bet and reduces IFN-γ, granzyme B, and perforin, impairing cytotoxic activities. Therefore, TUDCA restores CD8^+^ T-cell effector function by lowering ER stress and CHOP expression level [52]. In dust mite-induced asthma models, TUDCA has also been shown to reduce airway inflammation, mucous metaplasia, airway hyperresponsiveness, and fibrosis by suppressing pulmonary ER stress [83]. Importantly, both 4-PBA and TUDCA have been approved by the FDA for clinical use in metabolic diseases, including diabetes and obesity.

ISRIB, a small molecule that overcomes eIF2B inhibition caused by eIF2α phosphorylation, promotes the restoration of protein synthesis. In macrophages, phosphorylated eIF2α is essential for M2 polarization and metabolic reprogramming. Treatment of bone marrow-derived macrophages (BMDMs) or tumor-infiltrating macrophages with ISRIB suppresses the expression of M2 markers (e.g., Arg1), reduces oxygen consumption rate (OCR) and fatty acid oxidation (FAO) to reprogram macrophages towards a proinflammatory M1-like fate that promotes anti-tumor activities [53]. By contrast, Salubrinal, a stabilizer of eIF2α phosphorylation, enhances IFN-γ production via NF-κB activation and inhibits transmissible gastroenteritis virus (TGEV) replication [80].

### 3.6. Modulating Stress Granules in Immune Cells

In addition to transcriptional reprogramming, the UPR, particularly the PERK–eIF2α axis, exerts rapid and reversible control over immune cell function by remodeling the translational landscape. A key downstream consequence of eIF2α phosphorylation is the assembly of stress granules (SGs) due to the accumulation of untranslated messenger ribonucleoproteins (mRNPs). These “exposed” untranslated mRNAs interact with proteins containing low-complexity and intrinsically disordered domains (LCDs/IDRs) and undergo liquid–liquid phase separation (LLPS) to condense RNA, translation apparatus and signaling molecules into membraneless SG compartments [95,96,97]. ISRIB significantly suppresses SG formation by restoring bulk mRNA translation [98]. Although non-translating mRNAs are preferentially recruited to SGs, it is worth noting that recent studies demonstrated that translation of mRNAs localized in SGs is frequently observed [99,100,101]. The dynamic nature of SGs enables regulation of mRNA localization, translation, and degradation, and also serves as a hub for the assembly of signaling complexes involved in diverse immune responses.

During viral infection, phosphorylation of eIF2α is rapidly induced to promote SGs formation and halt viral protein translation—an antiviral mechanism that precedes the transcriptional activation of interferon-stimulated genes. Thus, SG assembly represents an important first-line innate immune defense mechanism [102]. However, many viruses have evolved to encode proteins that counteract this highly conserved response by preventing eIF2α phosphorylation or disrupting SGs assembly through degradation of core SG proteins [103].

In macrophages, SGs have been shown to sequester inflammatory cytokine mRNAs such as *TNFA* and *IL6*, by recruiting RNA-binding proteins like TIA-1 and TIAR, thereby transiently suppressing their translation and delaying the onset of inflammation [104]. This serves as a protective mechanism to restore homeostasis after initial immune activation by preventing excessive tissue damage.

In T cells, stress-induced SGs have been shown to dynamically regulate protein synthesis programs required for T cell activation and differentiation. Key SG components, such as G3BP1 and TIA-1, control mRNA accessibility and modulate the PI3K/AKT/mTOR pathway, influencing T cell polarization toward distinct lineages (e.g., Th1, Th2) [105]. Upon stimulation of resting human CD4^+^ and CD8^+^ T cells, SG constituents G3BP1, TIA-1, EIF4G1, and ELAVL1 are rapidly upregulated and coalesce into SGs, contributing to downregulation of immune checkpoint (IC) (e.g., PD1, LAG3) protein expression [106]. SGs also play a regulatory role in natural killer (NK) cells, where they sequester mRNAs encoding cytotoxic effectors such as granzyme B, perforin, and IFN-γ, thereby suppressing their expression to promote chronic inflammation and persistent viral infections [107,108].

**Figure 3:**
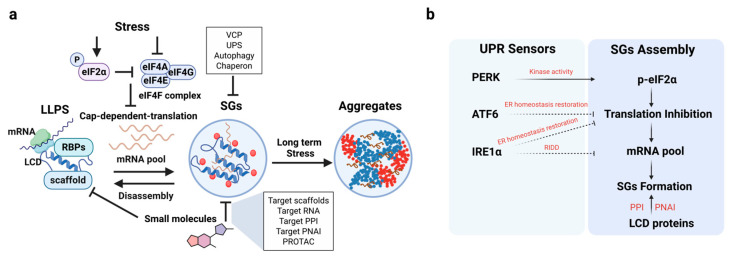
Integrated regulation of LCD-containing proteins, stress granules, and UPR signaling. (**a**): Under stress, phosphorylation of eIF2α disrupts eIF4F complex formation, suppressing translation initiation, leading to accumulation of untranslated mRNAs that associate with LCD-harboring RBPs. Through LLPS, these mRNP complexes condense into SGs, which dynamically assemble and disassemble to regulate mRNA translation, localization, and degradation. Persistent or unresolved stress drives SGs toward aggregate formation that is associated with pathological protein inclusions. SGs turnover is mediated by valosin-containing protein (VCP), ubiquitin–proteasome system (UPS), autophagy, and molecular chaperones. Small molecules can modulate SGs dynamics by targeting LCD-mediated scaffolds, RNA, PPI, or Protein–Nucleic acids interaction (PNAI). (**b**): Among the three canonical UPR sensors, PERK directly promotes SGs formation through its kinase activity by phosphorylating eIF2α, leading to translational inhibition, accumulation of untranslated mRNAs, and subsequent SGs assembly. In contrast, ATF6 and IRE1α primarily contribute to the restoration of ER homeostasis, thereby indirectly modulating the magnitude and duration of translational repression. In addition, IRE1α-mediated regulated RIDD can reshape the cellular mRNA pool, potentially influencing SGs composition. The assembly of SGs further relies on multivalent PPI and PNAI mediated by LCD-containing proteins.

Collectively, SGs function as dynamic regulatory hubs that integrate translational control with immune signaling across diverse immune cell types under various stress conditions. Pharmacological modulation of SGs formation or dissolution, therefore, offers a promising strategy to reshape immune responses in infection, inflammation, and cancer (Figure 3).

### 3.7. Modulating Low-Complexity Domains for UPR and Immune Control

Kinases, channels, and receptors are common drug targets due to their disease association and well-defined ligand-binding pockets. However, many regulatory proteins—particularly transcription factors, which are frequent drivers of disease—are characterized by protein sequences with significantly lower amino acid diversity than would be expected by chance.

A critical factor in targeting LCDs is their lack of a defined structure, unlike that of globular protein domains. However, it is important to note that a disordered protein sequence does not mean it is completely unstructured.

Small molecules have been identified to bind LCDs of several transcription factors, including p53 [109], EWS-FLI1, MYC, and nuclear receptors [110,111,112,113,114,115]. This suggests that LCDs are not entirely “undruggable.” Furthermore, with our increasing understanding of the conformational heterogeneity of LCDs and the advancement of molecular simulation and machine learning to predict LCD structures, we expect new opportunities in drug design. In particular, the variety of conformations an LCD can adopt should increase the number of potential high-affinity binders. This contrasts with folded protein targets where their relatively rigid structures provide only a few optimal binding solutions. Small molecules can potentially “trap” specific LCD conformations via induced fit, thereby altering protein function and specificity. As a proof-of-concept, using machine learning-based approaches, novel synthetic peptides targeting the LCD of G3BP1 were obtained and shown to block G3BP1’s ability to nucleate SG formation in response to oxidative stress [116]. However, it is unclear whether similar approaches can be employed to design small-molecule LCD binders with the requisite specificity and affinity. Finally, how to use these LCD modifiers to precisely target pathological LCD-mediated interactions without grossly impacting their normal function remains a formidable challenge.

The LCDs are abundantly found in SG constituents (e.g., TIA-1 [117], TDP-43 [118], hnRNPs [119]), UPR sensors (IRE1α and PERK [120,121]), immune sensors (e.g., cGAS [122]), and inflammasome (NLRP6 [123]). LCD-driven interactions and protein condensation play critical roles in stress and immune regulation [124]. As such, these LCDs represent a largely untapped proteome for small-molecule immune modulation.

Collectively, low-complexity domains represent structurally dynamic regulatory modules that influence stress response and immune function through protein condensation. Understanding the druggability of LCDs offer a new frontier in precision immunomodulatory drug design.

## 4. Discussion

The UPR signaling outputs are shaped by immune response context, signal intensity and duration, and the differentiation/activation state of the immune cells. This is particularly evident for IRE1α, whose RNase activities can generate adaptive signaling through XBP1 splicing, but can also trigger RIDD to degrade a subset of mRNAs and microRNAs—an output frequently linked to inflammatory reprogramming and cell death. What is clear is that IRE1α engages both *XBP1* splicing and RIDD, and that the balance between these two signal outputs can drive divergent and at times opposing biological outcomes [125,126,127]. This is not because IRE1α lacks specificity, but because its substrate specificity is strongly influenced by the cellular state and stress dynamics. What remains unclear is how RIDD substrates are selected in different immune settings. Future studies will need to both identify RNA features and cellular conditions that subject certain transcripts to RIDD. For example, metabolic RNA labeling strategies (e.g., SLAM-seq or 4sU-seq) can distinguish accelerated RNA decay from transcriptional repression [128]. In parallel, RNA structure–profiling methods, such as SHAPE- or DMS-based sequencing, may help uncover sequence or structural determinants that confer sensitivity to RIDD [129].

Distinct immune cell subsets exhibit marked differences in their sensitivity to and dependence on the UPR, leading to complex and highly cell-type-specific immunological effects when the UPR is perturbed. In DCs, the IRE1α–XBP1 axis regulates antigen processing and the maturation of MHC class I and II presentation pathways [130]. By contrast, in TAMs and MDSCs, UPR signaling reinforces their immunosuppressive functions. For example, sustained activation of XBP1 drives the production of VEGF and IL-10 in TAMs, thereby facilitating tumor immune evasion and progression [50]. In T cell subsets, the PERK–eIF2α pathway plays a key role in orchestrating T cell exhaustion programs [131], and in the development of CD8^+^ memory T cells [132]. Collectively, the UPR plays a dual role in immunity—capable of both enhancing anti-tumor responses and promoting immune suppression. This complexity poses significant challenges for the design of immunotherapeutic strategies, especially at the intersection of cancer and autoimmune diseases.

Despite a growing number of drugs that target the UPR for therapeutic purposes, current pharmacological strategies face important limitations that must be critically considered. ATP-competitive inhibitors targeting the UPR sensors, such as IRE1α and PERK, frequently exhibit off-target effects due to the conserved structures of the ATP-binding pockets across the kinome. Similarly, the IRE1α kinase inhibitor KIRA6 has been shown to also bind HSP60, disrupting mitochondrial integrity [133]. Although GSK2606414 is a highly selective inhibitor of PERK, its clinical utility is limited by significant on-target pancreatic toxicity [134]. Moreover, persistent inhibition of the UPR may impair its essential function in adaptive stress responses, particularly in secretory or metabolically active tissues, thus increasing the risk of systemic toxicity.

In contrast, indirect modulators, such as chemical chaperones (e.g., 4-PBA, TUDCA) or ISR pathway regulators (e.g., ISRIB), provide broader modulation of ER proteostasis but often lack pathway specificity that affects both cytoprotective and pathogenic functions of the UPR. For instance, ISRIB, while effective in reprogramming immune cells and modulating stress granules, is expected to also exert broad effects on global protein translation. This raises concerns about pleiotropic and off-target effects that could compromise immune cell function. Likewise, Sigma-1 receptor (Sig-1R) antagonists such as fluvoxamine, originally developed as antidepressants [135], preferentially engage the central nervous system due to its high Sig-1R expression [136]. Their use in non-central nervous system must carefully consider potential adverse effects on the brain and spinal cord.

Although numerous small-molecule UPR modulators have shown promise in reshaping immune responses, their clinical translation is often hampered by challenges in delivery efficiency and cellular targeting, particularly within complex immune microenvironments. Effective immune modulation requires pathway specificity and spatial precision. This is especially critical in diseases where immune cell subsets exhibit distinct thresholds for UPR activation or molecular basis of UPR dysregulation. To overcome these limitations, nanomaterial-based targeted drug delivery systems (TDDS) are emerging as a promising approach. These systems can deliver UPR modulators to specific subcellular compartments—such as the ER or SGs. For instance, celastrol-loaded FAP-α/ER dual-targeted liposomes, representing a lipid-based nanoparticle delivery system, achieve disease-site specificity and ER localization, thereby inducing localized ER stress-mediated apoptosis and suppressing synovial inflammation [137]. However, the ubiquitous expression of ER-resident sensors may lead to drug delivery to the wrong tissue or cell type. Beyond single-pathway modulation, a self-assembled TPP–PEG–biotin nanoparticle system has been reported to co-target GRP78 and lysosomes, resulting in PERK- and IRE1α-dependent UPR activation coupled with lysosomal disruption [138]. Furthermore, advanced nanotechnologies have been developed to mimic phase separation mechanisms of SGs, allowing for the targeted disruption of LCD-mediated condensates in the UPR pathway. DNA nanomechanical devices, owing to their programmability and spatial resolution, are increasingly viewed as artificial signal switches capable of dynamically regulating UPR pathways with subcellular precision [139].

Combining small-molecule inhibitors targeting the UPR with immune checkpoint inhibitors (ICIs) holds promise for overcoming immune resistance in cancer therapy. Co-administration of the IRE1α RNase inhibitor ORIN1001 with anti–PD-1 antibodies significantly enhanced CD8^+^ T cell infiltration and effector function in a triple-negative breast cancer model [126]. Similarly, the PERK inhibitor GSK2606414 improved T cell function and cooperated with PD-1 blockade to boost antitumor immunity in an immunogenic sarcoma model [140]. These findings suggest that selective UPR inhibition within immune-suppressive cell types in the TME—such as myeloid cells or exhausted T cells—can relieve intrinsic stress-related dysfunction, thereby amplifying ICI efficacy. In the future, AI-driven multi-omics analysis will likely identify tumors characterized by “UPR-high, immune-suppressive” signatures, enabling the development of personalized combination therapies that target both tumor-intrinsic stress pathways and immune evasion mechanisms.

Beyond cancer immunotherapy, severe viral infections such as SARS-CoV-2 are also associated with aberrant activation of the IRE1α–XBP1 branch of the UPR in myeloid cells. Pharmacological inhibition of IRE1α RNase activity effectively blunts this hyperinflammatory response [72]. Emerging evidence from neuroinflammatory and autoimmune models indicates that UPR signaling is activated in Parkinson’s disease and multiple sclerosis, leading to aberrant microglial activation or pathogenic T cell differentiation [141]. Despite compelling disease associations, clinical translation of UPR-targeted therapies remains constrained by several unresolved challenges. In addition to challenges in deciphering cell type-specific and context-dependent regulation of the UPR, clinically actionable biomarkers capable of resolving the various UPR states within specific immune subsets are lacking. Key UPR readouts such as *XBP1* splicing and eIF2α phosphorylation are difficult to standardize in patient samples. Finally, long-term safety represents a critical concern in treating chronic inflammatory diseases, as sustained inhibition of core UPR sensors, particularly PERK, causes metabolic toxicity that contributes to type 2 diabetes [142]. A deeper fundamental understanding of the regulation of the UPR in disease-relevant contexts is required to overcome current therapeutic bottlenecks.

## Figures and Tables

**Table 1:** Mechanisms and immune outcomes by targeting UPR signaling pathways with small molecules.

Small Molecule	Target	Strategy	Function	Clinical Development Status	Refs.
3-Ethoxy-5,6-dibromosalicylaldehyde	IRE1α	Inhibit RNase	Suppress XBP1 splicing and downstream pro-viral transcriptional programs	Preclinical	[69]
APY29	IRE1α oligomerization/kinase domain	Modulates oligomerization	Enhance RNase signaling and bias macrophage polarization toward an anti-inflammatory state	Preclinical	[70]
Compound 3	IRE1α oligomerization/kinase domain	Modulates oligomerization	Attenuate RNase activity and suppress pro-inflammatory cytokine expression	Preclinical	[70]
HA15	IRE1α–BiP interaction interface	PPI agonist	Activate IRE1α signaling and enhance antigen presentation and T cell priming	Preclinical	[71]
IXA4	IRE1α RNase domain	Selective RNase agonist	Activate XBP1 splicing to restore mitochondrial fitness and metabolic homeostasis	Preclinical	[72]
KIRA6	IRE1α kinase/ATP-binding domain	Kinase-inhibiting RNase attenuator (KIRA)	Dampen RNase activity and restrain pathological inflammatory cytokine production	Preclinical	[73]
KIRA8	IRE1α kinase/ATP-binding domain	Kinase-inhibiting RNase attenuator (KIRA)	Suppress RNase signaling and protect tissues from autoimmune T cell-mediated damage	Preclinical	[74]
Kaempferol	IRE1α–TRAF2 interaction	PPI disruption	Uncouples IRE1α–TRAF2 signaling to suppress JNK-driven inflammation	Preclinical	[75]
MKC3946	IRE1α RNase domain	Direct RNase inhibition	Suppress XBP1-dependent cytokine amplification and immune hyperactivation	Preclinical	[76]
STF-083010	IRE1α RNase domain	Direct RNase inhibition	Block XBP1 splicing to attenuate inflammasome-driven inflammation and T cell exhaustion	Preclinical	[77]
AMG44	PERK kinase domain	ATP-competitive kinase inhibition	Restrain pathogenic Th1/Th17 differentiation and mitigate T cell-driven GVHD	Preclinical	[78]
GSK2606414	PERK kinase domain	ATP-competitive kinase inhibition	Disrupt NRF2-dependent immunosuppressive programs and unleash STING-driven inflammatory responses	Clinical terminated	[79]
GSK2656157	PERK kinase domain	ATP-competitive kinase inhibition	Enhance anti-tumor immunity and checkpoint blockade efficacy	Clinical terminated	[53]
ISRIB	eIF2B	Inhibit eIF2α activity	Shutdown translation	Preclinical	[53]
Salubrinal	eIF2α dephosphorylation	Active eIF2α activity	Prolong the stress-adaptive translational program	Preclinical	[80]
AEBSF	S1P protease	Protease inhibition	Suppress ATF6-driven stress responses required for efficient viral replication	Preclinical	[81,82]
Ceapin-A7	ATF6–chaperone complex	Stabilizes inhibitory PPI	Prevent ATF6 transfer to Golgi and dampen ATF6-dependent inflammatory transcription	Preclinical	[83]
4-PBA (4-phenylbutyrate)	ER proteostasis	Chemical chaperone	Improves ER protein folding capacity	FDA-approved	[84]
Azoramide	ER proteostasis	ER proteostasis enhancer	Enhances ER folding and calcium homeostasis	Preclinical	[85]
TUDCA	ER proteostasis	Chemical chaperone	Relieve ER stress and suppress downstream pro-apoptotic and pro-inflammatory UPR outputs across branches	FDA-approved	[52,86]
Fluvoxamine/SA4503	Sigma-1 receptor	UPR tuning via S1R agonism	Relieve ER stress and suppress downstream pro-apoptotic and pro-inflammatory UPR outputs across branches	Fluvoxamine: FDA-approved, SA4503: Clinical trials (Phase II)	[87]

**Table 2:** Comparative strategies for pharmacological targeting of distinct UPR branches and protein domains.

Drug Target	Advantage	Limation
IRE1α RNase domain	Directly blocks XBP1 splicing and RNase-dependent outputs	Pharmacologically difficult to separate adaptive XBP1s from pro-death RIDD
IRE1α/PERK ATP kinase domain	Enables conformational control via the kinase pocket	Limited by kinome selectivity and exposure-dependent toxicity
IRE1α/PERK/ATF6 oligomerization	Modulates activation threshold and signaling amplitude	Dynamic protein–protein interfaces limit potency and druggability
PPIs of IRE1α/PERK/ATF6	Rewires downstream signaling without abolishing core enzymatic activity	Weak and transient PPIs pose major druggability challenges
Chaperones	Simultaneously affects multiple UPR arms	Narrow therapeutic window due to essential proteostasis functions and client variety
Stress granules	Rapid and reversible control of translational programs	Pleiotropic effects, complicating target validation
Low-complexity domains	Disrupt transcriptional or signaling condensates	High risk of off-target effects and poor structural definition of condensates
Global ER proteostasis enhancers	Broadly reduce ER stress burden and restore folding capacity	Pleiotropic mechanisms with low pathway specificity

## Data Availability

No new data were created or analyzed in this study. Data sharing is not applicable to this article.
